# Response of *Lactobacillus plantarum* VAL6 to challenges of pH and sodium chloride stresses

**DOI:** 10.1038/s41598-020-80634-1

**Published:** 2021-01-14

**Authors:** Phu-Tho Nguyen, Thi-Tho Nguyen, Thi-Ngoc-Tuyen Vo, Thi-Thanh-Xuan Nguyen, Quoc-Khanh Hoang, Huu-Thanh Nguyen

**Affiliations:** 1 Graduate University of Sciences and Technology, Vietnam Academy of Science and Technology, Hanoi, Vietnam; 2 An Giang University, Vietnam National University, Ho Chi Minh City, Vietnam; 3 Hutech University, Ho Chi Minh City, Vietnam; 4 Tran Van Thanh High School, An Giang, Vietnam; 5 Institute of Tropical Biology, Vietnam Academy of Science and Technology, Hanoi, Vietnam

**Keywords:** Cell biology, Microbiology

## Abstract

To investigate the effect of environmental stresses on the exopolysaccharide biosynthesis, after 24 h of culture at 37 °C with pH 6.8 and without sodium chloride, *Lactobacillus plantarum* VAL6 was exposed to different stress conditions, including pH (pHs of 3 and 8) and high sodium chloride concentration treatments. The results found that *Lactobacillus plantarum* VAL6 exposed to stress at pH 3 for 3 h gives the highest exopolysaccharide yield (50.44 g/L) which is 6.4 fold higher than non-stress. Under pH and sodium chloride stresses, the mannose content in exopolysaccharides decreased while the glucose increased in comparison with non-stress condition. The galactose content was highest under stress condition of pH 8 meantime rhamnose content increased sharply when *Lactobacillus plantarum* VAL6 was stressed at pH 3. The arabinose content in exopolysaccharides was not detected under non-stress condition but it was recorded in great amounts after 3 h of stress at pH 3. In addition, stress of pH 8 triggered the mRNA expression of *eps*F gene resulting in galactose-rich EPS synthesis. According to our results, the stresses of pH and sodium chloride enhance the production and change the mRNA expression of *eps*F gene, leading to differences in the monosaccharide composition of exopolysaccharides.

## Introduction

Exopolysaccharides (EPS) are high molecular weight biological polymers synthesized extracellularly by various microorganisms (archaea, bacteria, fungi or algae) and applied in the variety of industries such as food, pharmaceuticals, etc.^1^. Especially, Lactic acid bacterial (LAB) are the most remarkable group of EPS producing bacteria because of their strong production of EPS^2^ and they are GRAS (Generally Recognized As Safe)^3^. Dextran, levan, oligosaccharides, etc. are polysaccharides produced by LAB and widely used in the food industry to improve the rheological, structural and sensory properties of fermented products^2^. In addition, LAB's EPS can also possess biological functions such as immune stimulation, antitumour effects, prebiotic activities and antioxidants^4^.

The function of EPS for bacteria is to protect cells from the negative effects of environment as dehydration, antibiotics, phagocytosis and phage attacks because of their role in forming biofilms^5–7^. Changes in environmental conditions may alter the EPS production^8^ and result in the biosynthesis of new types of EPS in bacteria^9^. The final EPS yield and the characteristics of EPS are strongly influenced by environmental conditions^10,11^. Fedorová et al.^12^ have found a positive correlation between EPS production and *L. reuteri* resistance to low pH stress. Seesuriyachan et al.^13^ also reported that EPS production increased when *L. confusus* was stressed under high salinity. Moreover, the effect of various environmental conditions can trigger the expression of genes involved in the biosynthesis of EPS in LAB. For example, when the pH of the culture medium decreased from pH 6.5 to pH 5.5, the *eps* gene cluster expression increased in *S. thermophilus* ASCC 1275^14^.

The EPS biological properties are determined by its monosacharide composition. The differences in the ratio and composition of monosaccharides cause the alteration of biological activity and therapeutic effect^15^. For instance, the anti-inflammatory activity of EPS is related to the sugar ratio of galactose, rhamnose and glucose. Galactose-rich EPS enhances their anti-inflammatory activity^16^. Rare sugar-rich EPS have a higher biological activity than EPS consisting of common sugars. Some of rare sugars as L-fucose, L-rhamnose, and uronic acid contain valuable properties which maybe attractive to a large number of applications for various fields including antioxidant, anti-inflammatory substances, etc.; particularly, such rare sugars can be used to synthesize the nucleoside substances which have antiviral effects^17^. Rhamnose-rich EPS secreted by *L. paracasei* have ability to boost the immune system^18^. Bacteria can produce EPS containing rare sugars under certain conditions^19^. The EPS synthesized by LAB can contain both common and rare sugars, depending highly on environmental conditions^20^. The monosaccharide composition of EPS also involve LAB’s resistance to stress. According to the study result of London et al.^21^, the stress resistance of *L. mucosae* DPC 6426 depended on the monosaccharide ratio of EPS produced by themself. Furthermore, the proportions of individual monosaccharides in EPS differed when *L. delbrueckii* subsp. *bulgaricus* CNRZ 1187 and *L. delbrueckii* subsp. *bulgaricus* CNRZ 416 were cultured under various pH conditions^22^.

Environmental stresses can alter biosynthesis and metabolism in microorganisms^23^. Although there are many reports on the relationship between changed environmental conditions and EPS biosynthesis, studies on the effects of environmental stress on EPS synthesis in Lactobacilli are still limited. *L. plantarum* has been known to be a producer of EPS with distinct biological properties and activities for different applications, including health and food industry^24^. Thus, this study focuses on assessing the impact of stress conditions such as pH (low and high) and high sodium chloride concentration on EPS synthesis in *L. plantarum* VAL6. The purpose of this work is to improve the yield and monosaccharide composition of EPS for highly bioactive EPS production.

## Results

### Effect of pH and sodium chloride stresses on EPS production

**Figure 1:**
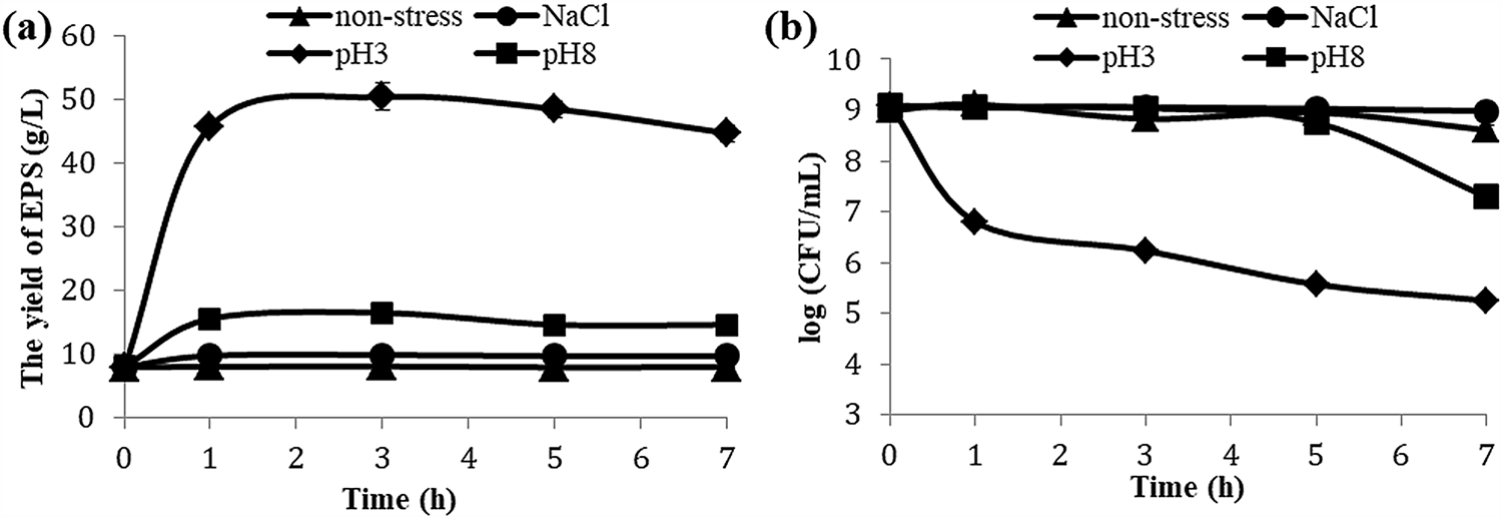
The EPS yield (**a**) and cell density (**b**) of *L. plantarum* VAL6 under pH and sodium chloride stresses. The non-stress control was maintained at 37 °C, pH 6.8 and without sodium chloride for the entire time. Standard deviation is displayed and account for three independent experiments.

The harsh environmental conditions stimulate microorganisms to develop various adaptive strategies, allowing them to cope with the adverse effects of extreme conditions. Among these strategies, EPS biosynthesis is one of the most common protection mechanisms^11^. We wonder if it is true during *L. plantarum* VAL6′s EPS production under pH and sodium chloride stresses. Therefore, *L. plantarum* VAL6 was treated with stress conditions of pH 3, pH 8 and high concentration of sodium chloride. As results of our data, EPS yield increases considerably under stress treatments of pH 3 and pH 8 compared to non-stressed condition (Fig. 1a).

The amount of EPS produced by *L. plantarum* VAL6 under stress of pH 3 was higher than that under other stress conditions. The highest EPS yield produced by ones was over 50 g/L after exposure to stress at pH 3 for 3 h and 6.4 fold higher than the control (only 7.8 g/L). While under stress at pH 8, the yield of EPS was about 16 g/L and 2.1 fold higher. These results indicated that *L. plantarum* VAL6 enhances EPS synthesis under stress conditions of pH 3 and pH 8.

The production of EPS was influenced by not only pH stress but also sodium chloride stress. The effect of sodium chloride stress on EPS production in *L. plantarum* VAL6 was determined in the presence of sodium chloride (10%). It is observed that there is a slight increase in the production of EPS compared to non-stress (Fig. 1a). Under sodium chloride stress, the yield of EPS fluctuated approxymately 10 g/L. However, there was still a significant difference (*P* < 0.05) in EPS yield between sodium chloride stress and non-stress.

In addition to the evaluation of EPS production, the growth capacity of *L. plantarum* VAL6 under stress conditions was also examined. The results showed that *L. plantarum* VAL6 is able to survive during the time of tested stresses (Fig. 1b). Under the stress conditions of NaCl and pH 8, the density of *L. plantarum* VAL6 was found an insignificant difference (*P* > 0.05) compared to non-stress. However, the density of *L. plantarum* VAL6 decreased strongly under stress condition of pH 3.

### The changes in the monosaccharide compositions of EPS

**Figure 2:**
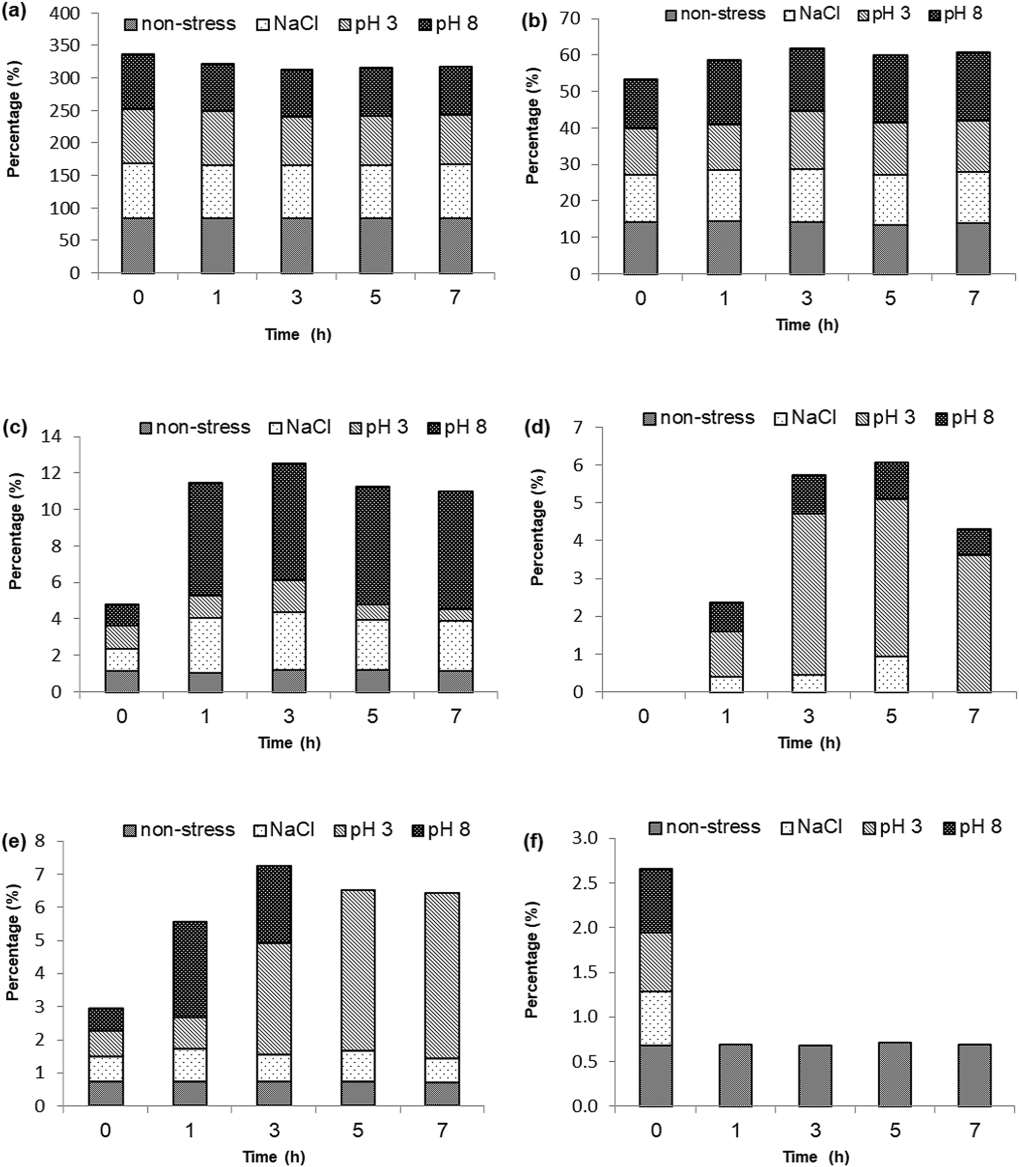
Proportions of monosaccharides in the EPS produced by *L. plantarum* VAL6 under pH and sodium chloride stresses: (**a**) mannose; (**b**) glucose; (**c**) galactose; (**d**) arabinose; (**e**) rhamnose; (**f**) xylose. The non-stress control was maintained at 37 °C, pH 6.8 and without sodium chloride for the entire time.

Environmental conditions greatly affect the yield, monosaccharide composition as well as functional properties of EPS synthesized by bacteria^10,11^. To better clarify the effect of environmental conditions on EPS biosynthesis, we have evaluated the impact of pH and sodium chloride stress conditions on changes in the monosaccharide composition of EPS. The results were shown in Fig. 2.

The ratio of monosaccharides in EPS differently fluctuated between non-stress and stress conditions. Stress conditions decreased the ratio of mannose in EPS (Fig. 2a). Under stress of sodium chloride, the percentage of mannose fluctuated around 81% during the time of stress and was lower than non-stress samples (84.33%). For stress of pH 3, the percentage of mannose was 83.46% after 1 h of stress but then dropped to 75–76% when extending the stress period. For stress of pH 8, the proportion of mannose in EPS was only 73–74%.

In contrast, stress conditions increased glucose levels (Fig. 2b). Under stress of pH 3, the glucose content in EPS increased from 12.55% after 1 h of stress to a maximum ratio of 15.77% after 3 h and then it steadily decreased to 14.21% after 7 h of stress. Whereas under stress of pH 8, the glucose ratio fluctuated between 17.15 and 18.59%. The glucose content in EPS under sodium chloride stress increased slightly from 13.88% after 1 h of stress to a approximate maximum of 14.7% at 3 h and then it steadily decreased according to the time of stress. These compared with about 13% under non-stress condition.

The galactose ratio in EPS produced by *L. plantarum* VAL6 in exposure to stress at pH 8 sharply increased (Fig. 2c). Under stress of pH 8, the galactose ratio ranged 6.20–6.49% compared to 1.21% of the control. This value was only about 0.62–1.75% under stress of pH 3. It was also observed that galactose ratio in EPS slightly increased when *L. plantarum* VAL6 was exposed to sodium chloride stress and ranged 2.7–3%.

Research results also indicated that there was a difference in the rhamnose content of EPS when *L. plantarum* VAL6 was exposed to pH stress conditions (Fig. 2e). Especially, the rhamnose content of EPS from *L. plantarum* VAL6 stressed at pH 3 was highest and increased gradually following to the time of stress. It was approximately 5% after 7 h of stress, 6.6 times higher than non-stress. Under stress of pH 8, the ratio of rhamnose fluctuated approximately 2% and it was did not detected during stress periods over 3 h. For sodium chloride stress, the ratio of rhamnose was about 1%.

Arabinose was not detected in EPS composition under non-stress condition, but it was found under tested stress conditions (Fig. 2d). Under stress of pH 3, the arabinose content in EPS was considerably higher than that under stress conditions of pH 8 and sodium chloride. The arabinose ratio was highest when *L. plantarum* VAL6 exposed to stress at pH 3 for 3 h. It peaked at 4.25% compared to just about 1% under stress conditions of pH 8 and sodium chloride. In contrast, xylose was found in the EPS composition of the controls although it was quite small. However, it was not detected in EPS produced by *L. plantarum* VAL6 under tested stress conditions (Fig. 2f).

### Expression of EPS genes

**Figure 3:**
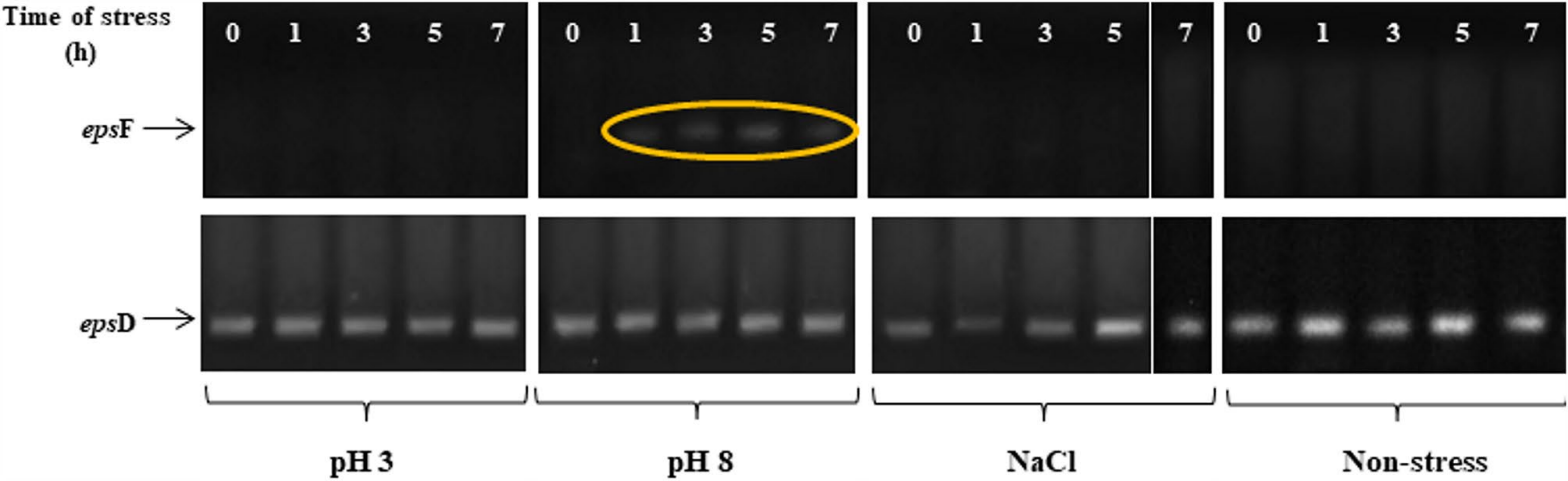
The expression of *eps*F and *eps*D gene under stress conditions of pH 3, pH 8, NaCl and non-stress. The non-stress control was maintained at 37 °C, pH 6.8 and without sodium chloride for the entire time. Full-length gels are presented in Supplementary Fig. S3.

Changes in expression of genes involved in EPS biosynthesis under different environmental conditions have been previously reported^25,26^. Assuming that alterations in the monosaccharide composition of EPS produced by *L. plantarum* VAL6 under pH and sodium chloride stresses could be also related to the expression of *eps* genes, we analyzed the expression of the two genes including *eps*D (encoding a putative protein tyrosine phosphatase) and *eps*F (encoding a putative glycosyltransferase)^27^ (see “Materials and methods” section for the primers used to amlify cDNA, Table 2). Results showed that *eps*D was expressed both stress and non-stress conditions (Fig. 3). Meanwhile, *eps*F was only expressed under stress of pH 8 (Fig. 3).

## Discussion

The results showed that stress of pH 3 stimulates *L. plantarum* VAL6 to strongly synthesize EPS. It is explained that EPS affect cell's sensitivity to acid pH. Anions linked to EPS can limit acid’s access to bacterial cells^28^. Moreover, cultures were stressed at low pH by addition of H_3_PO_4_. Therefore, having been synthesized, EPS may be attached to phosphate groups from H_3_PO_4_ by phosphorylation in order to form phosphate polysaccharide leading to increase the weight of obtained EPS. The presence of phosphate groups in EPS has been also recorded in some studies. EPS-b and EPS-r produced by *L. plantarum* EP56 contained phosphate groups which contribute to their negative charge^29^. Phosphate groups may provide important properties to EPS because they are required for the activation of lymphocytes^30^. The negative charge on EPS surface plays an important role in isolating positively charged toxic compounds such as nisin and metal ions^31^. In this study, phosphate rich EPS may be synthesized by *L. plantarum* VAL6 under stress condition of pH 3. Several studies also demonstrated that there is a correlation between low pH and EPS production. The contemporary pH decrease and the presence of sugar (osmotic stress) stimulated EPS production in LAB^32^. The EPS producing *L. mucosae* DPC 6426 exhibited a fivefold increasing survival during a 60-min exposure to HCl compared to EPS non-producing *L. mucosae* DPC 6420^21^. The strains of *L. reuteri* producing more EPS showed a considerably higher tolerance at low pH. The resistance of *L. reuteri* to the stress conditions of gastrointestinal tract was related to EPS production^12^. For treatment of pH 8, although EPS yield was lower compared to treatment pH 3, it was still higher than the control sample. This result indicated that *L. plantarum* VAL6 also enhances EPS synthesis to cope with the effects of high pH. There are few studies reporting on relationships between alkaline pH and EPS production. However, it was found that EPS related to flocs formation, composed of a complex mixture of EPS, provide microorganism protection against alkaline pH^33^.

The high concentration of sodium chloride affected positively EPS production in *L. plantarum* VAL6. The increase in EPS production was related to sodium chloride tolerance^34^. The extracellular polysaccharide layer with high water retention helps microorganisms for better resistance to osmosis^35^. Another study has also demonstrated that *L. confusus* TISTR 1498 overproduced EPS under high salinity stress^13^. The effect of sodium chloride on the biosynthesis of EPS in LAB has been little reported. However, many studies on cyanobacteria demonstrated that high sodium chloride concentration positively affects EPS production. There was a considerable increase in EPS production of *Synechocystis* sp. exposed to sodium chloride^34^. The production of extracellular carbohydrates by *Microcoleus vaginatus* also considerably increased under higher salt concentrations^36^.

The results of growth kinetics demonstrated that *L. plantarum* VAL6 is able to survive during tested stresses. Despite a sharp decrease, the density of *L. plantarum* VAL6 was still higher than 5 log CFU/mL after 7 h of stress at pH 3. Many studies have also demonstrated that *L. plantarum* can outlive under extremely low pH condition. According to the study of Lee et al.^37^, some *L. plantarum* strains isolated from Korean kimchi could survive at pH 2.5 for 2 h. The density of *L. plantarum* ZDY 2013 altered inconsiderably at pH 3 and could survive for 6 h at pH 2 at over 7 log CFU/mL^38^.

This research characterised monosaccharides making of EPS produced by *L. plantarum* VAL6 with and without exposure to pH and sodium chloride stresses, then quantified the monosaccharide compositions by GC-FID. Overall, monosaccharide composition of EPS produced by *L. plantarum* VAL6 was composed of different monomers. The presence of mannose, glucose and galactose was common in both non-stress and stress conditions. The EPS of *L. plantarum* VAL6 from non-stress samples was found to be comprised of five identifiable monosachharides. In descending order, mannose, glucose, galactose were three major monosaccharides present in these samples. This result was not the same as other *L. plantarum* species studied previously such as *L. plantarum* EP56^29^, *L. plantarum* C88^39^, *L. plantarum* YW11^40^, where glucose was main component sugar. However, the monosaccharide ratio of EPS produced by *L. plantarum* VAL6 was similar to the monosaccharide ratio of EPS produced by *L. plantarum* 70810 (18.21 Glc: 3.03 Gal: 78.76 Man)^41^. Likewise, mannose, glucose and galactose (in a molar ratio of 12.94:7.26:3.31) were the main composition of EPS produced by *L. plantarum* KX041^15^.

The monosaccharide composition of EPS from *L. plantarum* VAL6 exposed to tested stress treatments changed both monosaccharide type and the ratio of sugars. This result suggested that stresses of pH and sodium chloride can trigger the reprogramming of cellular mechanism for EPS biosynthesis resulting in changes in monosacchride composition of EPS with the accumulation of certain common and rare sugar ratios (Table 1). Remarkably, the result found a substantial increase in rhamnose content under stress condition of pH 3. Rhamnose-rich EPS show interesting biological activities which make them likely to be used for many applications such as pharmaceuticals, cosmetics and functional foods^42,43^. For instance, rhamnose and fucose have gotten more attention when oligo and polysaccharides rich in rhamnose and fucose were found to be associated with anti-aging skin mechanisms^44^. Also according to this study, galactose content in EPS increased sharply when *L. plantarum* VAL6 was exposed to stress of pH 8. As reported by previous studies, galatose-rich EPS could enhance anti-inflammatory activity^16^. Galactose-rich EPS also increased the adhesion and capacity of biofilm formation in LAB^45^. Another interesting result found in our study was the presence of arabinose in EPS under tested stresses but it was not detected under non-stress condition. This result suggested that arabinose component in EPS may be involved in bacterial stress resistance. A prior study has also demonstrated that the arabinose component in EPS plays an important role in cell aggregation to deal with hostile environmental conditions^46^. These results elicit the using potential of pH stress for the production of carbohydrate bioactive compounds.

**Table 1:** Summary of the changes in the monosacharide composition of EPS produced by *L. plantarum* VAL6 under pH and sodium chloride stresses.

Monosaccharides	The changes in composition of monosacharides	
	Non-stress	Stress
Mannose	+	↓
Glucose	+	↑
Galactose	+	↑
Arabinose	−	+
Rhamnose	+	↑
Xylose	+	−

(−) Not detected; (+) Detected; (↓) Decreased; (↑) Increased.

The changes in the monosaccharide composition of EPS were related to the expression of tested *eps* genes. *eps*D gene takes part in the functional group which encodes enzymes involved in phosphoregulatory system, it controls polysaccharide assembly^20^. This may explain why *eps*D is always expressed in EPS synthesis. The results of this study also showed that *eps*D was expressed under non-stress and tested stress conditions (Fig. 3). However, *eps*F gene was only expressed under stress condition of pH 8 (Fig. 3). Furthermore, the result of study also demonstrated that stress of pH 8 strongly increased galactose content in EPS (Fig. 2c). In *S. thermophilus* Sfi6, *eps*F has been shown to encode a galactosyltransferase^47^, a transport enzyme which adds galactose to the repeating unit of EPS structure, leading to increase galactose proportion in the monosaccharide composition of EPS. Similarly, the results of this study suggested that the expression of *eps*F gene is related to the synthesis of galactose-rich EPS in *L. plantarum* VAL6.

## Conclusion

*L. plantarum* VAL6 was able to survive and enhance EPS production under pH and sodium chloride stresses. The highest yield of EPS reached to 50.44 g/L when *L. plantarum* VAL6 was exposed to stress of pH 3. The results also demonstrated that the stresses of pH 8 and sodium chloride positively affect the EPS producing ability of *L. plantarum* VAL6*.* Futhermore, the monosaccharide compositions in EPS synthesized by *L. plantarum* VAL6 differently changed under the impact of these stresses. Under tested stress conditions, the content of mannose decreased while glucose content increased compared to non-stress circumstance. Galactose content dramatically increased when *L. plantarum* VAL6 was exposed to stress of pH 8. There was an increase in the proportion of rare sugars (rhamnose and arabinose) when *L. plantarum* VAL6 was exposed to stress treatments. Rhamnose content in EPS was highest when *L. plantarum* VAL6 was stressed at pH 3 for 7 h. A large amount of arabinose was found in EPS when *L. plantarum* VAL6 was exposed to stress at pH 3 for 3 to 7 h. There was a relationship between *eps*F gene expression and high galactose content in EPS synthesized under stress of pH 8. These results elicit the biosynthesis of EPS in order to enrich rare sugars by using environmental stresses. The EPS with higher proportion of rare monosaccharides which is capable of increasing their biological properties can be used for different applications.

## Materials and methods

### Microbial strain, cultivation medium and stress conditions

*Lactobacillus plantarum* VAL6 (or *Lactiplantibacillus plantarum* VAL6, according to the taxonomy of the new genus Lactobacillus described by Zheng et al.^48^) was isolated from pickled vegetables. The strain was identified by molecular method using 16S rRNA sequence analysis and amplification to check the *rec*A gene fragment. The primers used for amplifying the 16S rRNA region were 27f. (5′-AGAGTTTGATCCTGGCTCAG-3′) and 1492r (5′-GGTTACCTTGTTACGACTT-3′) as forward and reverse primers, respectively^49^. The primers used for *recA* gene fragment amplification were planF (5′-CCG TTT ATGCGGAACACCTA-3′), and pREV (5′-TCGGGATTACCAAACATCAC-3′)^50^. The amplification of *recA* gene region Cultures were performed on Man–Rogosa–Sharpe medium (MRS)^51^. All the cultures have been carried out in a 5 L bioreactor (BIOSTAT, Sartorius Stedim Biotech GmbH, Germany). Briefly, 5 L of MRS medium was inoculated with 100 mL of overnight bacterial culture (OD_595_ = 1.5). pH was maintained to 6.8 (regulation by addition of NaOH 10 M), temperature was kept at 37 °C, agitation rate was set up to 250 rpm under aerobic facultative condition.

For pH stress, after 24 h of culture at 37 °C and pH 6.8, the culture was treated with pH conditions either pH 3 or pH 8 for 7 h. The time was calculated when the bioreactor reached the required pH. For sodium chloride stress, after 24 h of culture at 37 °C and pH 6.8, the culture was treated by adding NaCl to reach a concentration of 10% for 7 h. The non-stress control was simultaneously carried out in another bioreactor with pH 6.8 for the entire time. Under both stress and non-stress conditions, temperature was maintained at 37 °C and agitation rate was set up to 250 rpm. A volume of 500 mL of culture was sampled in every hour during the stress treatment for EPS quantification and characterization.

### EPS extraction and quantification

EPS was extracted from cell suspensions collected at the given time point according to the method described by Salazar et al.^52^ and Nguyen et al.^53^. Crude EPS total produced by *L. plantarum* VAL6 was harvested after 24 h by mixing 100 mL of supernatant with an equal amount of NaOH 2 M and was gently stirred overnight at room temperature. Supernatants were then recovered by centrifugation at 8400 g for 20 min and crude EPS was precipitated from the supernatants by adding twice the volume of 96% (v/v) cold ethanol. The precipitation was carried out at 4 °C for 48 h. After the second centrifugation step at 8400 g and 4 °C for 30 min, EPS was dried at 55 °C until constant weight. Consequently, the total EPS yield was determined gravimetrically by calculating the total weight of EPS per liter of culture medium.

### Compositional analysis of EPS by GC-FID

Monosaccharide composition of EPS was analyzed by GC-FID according to the method described by Yuan et al.^54^ with some modify^54^. 0.1 g of EPS was hydrolyzed with 3 mL of 2 M trifluoroacetic acid (TFA) at 105 °C for 4 h. The hydrolysed sugars were derivatised by the addition of hydroxylamine, pyridine and were subjected to acetylation using acetic anhydride (CH_3_CO)_2_O. The derivative products were used for determination of the monosaccharide composition by Gas Chromatography (GC). Sample of 1 μL was injected into the GC-FID using autosampler. GC was performed on Agilent 6890 N (USA) and HP-5MS UI column (30 m length 0.25 mm inner diameter 0.25 μm film thickness). Nitrogen was used as the carrier gas with 1 mL/min flow rate. The chromatographic conditions used are as follows: the initial column temperature was held at 120 °C for 2 min, increased at a rate of 20 °C/min to 200 °C for 4 min, and then subsequently increased at 25 °C/min to 280 °C, where it was held at 290 °C for 5 min. The comparison was made with standard mannose, glucose, galactose, rhamnose, arabinose, xylose and fucose for sugar identification.

### Examine the expression of eps genes

Total RNA was extracted from 500 μL cell suspension of *L. plantarum* VAL6 using TRIzol reagent (Invitrogen, UK) according to the manufacturer’s instruction. RNA was treated with RQ1 RNase-free DNase (Promega, Madison, USA) to remove contamination of chromosomal DNA. Qualitative test of RNA at 260 and 280 nm was found to be.

more than 1.8. One-Step RT-PCR was performed to synthesize cDNA from 1 μg of the DNase-treated RNA using HiSenScript RH(-) cDNA Synthesis Kit (iNtRON, Korea). Conventional PCR was done to amplify complementary DNA (cDNA). The reaction mixture (48 µL of master mix: 5 µL of 10X buffer, 0.5 µL of 10 mM dNTP mixture, 2 µL of 5 μM primers, 0.5 µL of Taq and 40 µL of distilled water) was mixed with 2 µL of template cDNA. The primers were shown in Table 2. The cycle conditions were as follows: 95 °C for 3 min; 30 cycles of 95 °C for 30 s, 58 °C for 30 s and 72 °C for 30 s; 72 °C for 5 min; and then maintenance at 25 °C for 1 min. The PCR products were checked the expression of *eps* genes by electrophoresis in 2% agarose gel.

**Table 2:** The primers used for the amplification^55^.

Gene	Primer	Sequence (5′ → 3′)	*T*_m_ (°C)	Product size (bp)
*eps*D	DF2 sense	TATTCTGGAGGCGTTTTTGG	60.1	205
	DR2 antisense	AAACTCACGGGCCATTTTT	59.4	
*eps*F	FF2 sense	TCAAGCGGGTATTTGACTTCTT	60.1	238
	FR2 antisense	AACATTGGGCCATCAACATC	60.6	

### Statistical analysis

The experiments were done three times. All the data were expressed as means ± standard deviations. Significance of difference was evaluated with one way ANOVA, followed by Tukey's HSD procedure to identify statistically significant differences at the 95.0% confidence interval. One-way analysis of variance was performed. Tukey's HSD multiple-range tests were applied to the individual variables to compare means and to assess if there was a significant difference.

## Supplementary Information

Supplementary Figure S3.
