# Supplementary material for: Response of Lactobacillus plantarum VAL6 to challenges of pH and sodium chloride stresses
Source: Sci Rep. 2021 Jan 14;11:1301. doi: 10.1038/s41598-020-80634-1 (PMC7809271; doi:10.1038/s41598-020-80634-1)
Supplement: Supplementary file 1 — Supplementary Figure S3. [file 41598_2020_80634_MOESM1_ESM.docx]

## Response of *Lactobacillus plantarum* VAL6 to challenges of pH and sodium chloride stresses on the exopolysaccharide synthesis

Phu-Tho Nguyen^1,2^, Thi-Tho Nguyen^3^, Thi-Ngoc-Tuyen Vo^4^, Thi-Thanh-Xuan Nguyen^2^, Quoc-Khanh Hoang^5^, Huu-Thanh Nguyen^2🖂^

^1^ Graduate University of Sciences and Technology, Vietnam Academy of Science and Technology, Vietnam.

^2^ An Giang University, Vietnam National University Ho Chi Minh City, Vietnam. ^🖂^Email: nhthanh@agu.edu.vn

^3^ Hutech University- Ho Chi Minh City, Vietnam.

^4^ Tran Van Thanh High School, An Giang, Vietnam.

^5^ Institute of Tropical Biology, Vietnam Academy of Science and Technology, Vietnam.


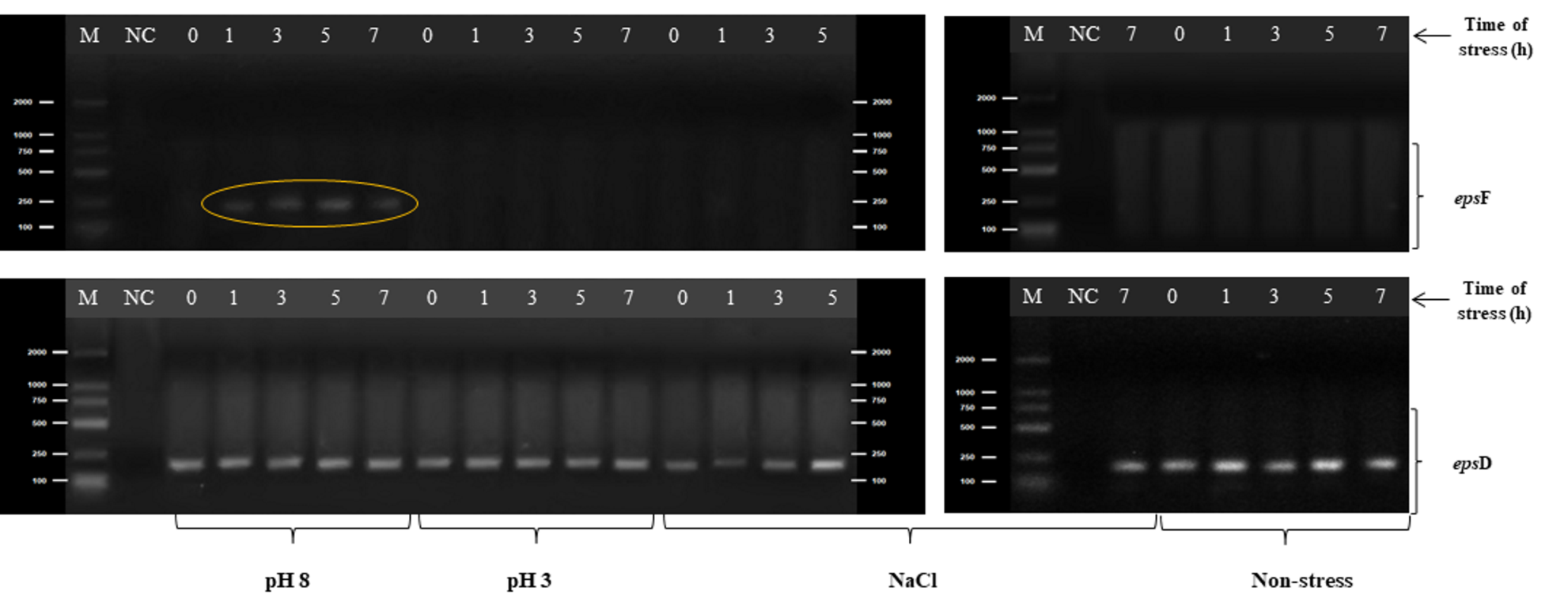


**Supplementary figure S3.** Full-length gels for the expression of *eps*F and *eps*D gene under stress conditions of pH 3, pH 8, NaCl and non-stress. M: DNA ladder; NC: Nagative control.
